# Short term sodium alendronate administration improves the peri-implant bone quality in osteoporotic animals

**DOI:** 10.1590/1678-77572016-0165

**Published:** 2017

**Authors:** Danila de OLIVEIRA, Jaqueline Suemi HASSUMI, Pedro Henrique da Silva GOMES-FERREIRA, Tárik Ocon Braga POLO, Gabriel Ramalho FERREIRA, Leonardo Perez FAVERANI, Roberta OKAMOTO

**Affiliations:** 1 Universidade Estadual Paulista, Faculdade de Odontologia de Araçatuba, Departamento de Ciências Básicas, Araçatuba, SP, Brasil.; 2 Universidade Estadual Paulista, Faculdade de Odontologia de Araçatuba, Departamento de Cirurgia e Clínica Integrada, Araçatuba, SP, Brasil.; 3 Universidade de São Paulo, Hospital de Reabilitação de Anomalias Craniofaciais, Bauru, SP, Brasil.

**Keywords:** Alendronate, Osseointegration, Osteoporosis

## Abstract

Sodium alendronate is a bisphosphonate drug that exerts antiresorptive action and is used to treat osteoporosis.

**Objective:**

The aim of this study was to evaluate the bone repair process at the bone/implant interface of osteoporotic rats treated with sodium alendronate through the analysis of microtomography, real time polymerase chain reactions and immunohistochemistry (RUNX2 protein, bone sialoprotein (BSP), alkaline phosphatase, osteopontin and osteocalcin).

**Material and Methods:**

A total of 42 rats were used and divided in to the following experimental groups: CTL: control group (rats submitted to fictitious surgery and fed with a balanced diet), OST: osteoporosis group (rats submitted to a bilateral ovariectomy and fed with a low calcium diet) and ALE: alendronate group (rats submitted to a bilateral ovariectomy, fed with a low calcium diet and treated with sodium alendronate). A surface treated implant was installed in both tibial metaphyses of each rat. Euthanasia of the animals was conducted at 14 (immunhostochemistry) and 42 days (immunohistochemistry, micro CT and PCR). Data were subjected to statistical analysis with a 5% significance level.

**Results:**

Bone volume (BV) and total pore volume were higher for ALE group (P<0.05). Molecular data for RUNX2 and BSP proteins were significantly expressed in the ALE group (P<0.05), in comparison with the other groups. ALP expression was higher in the CTL group (P<0.05). The immunostaining for RUNX2 and osteopontin was positive in the osteoblastic lineage cells of neoformed bone for the CTL and ALE groups in both periods (14 and 42 days). Alkaline phosphatase presented a lower staining area in the OST group compared to the CTL in both periods and the ALE at 42 days.

**Conclusion:**

There was a decrease of osteocalcin precipitation at 42 days for the ALE and OST groups. Therefore, treatment with short-term sodium alendronate improved bone repair around the implants installed in the tibia of osteoporotic rats.

## Introduction

Rehabilitation with dental implants has become widespread in society and most of the patients demanding this treatment are above 60 years old, a life stage in which osteoporosis is very common, especially in women^7^. Studies regarding dental implants in osteoporotic patients claim that the osteoporotic bone is similar to the proposed model of type IV bone, that is, residual bone formed by a thin layer of cortical bone surrounding the low density cancellous bone. This classification takes into consideration the bone quality, ranging from type I to IV^13^. Because of this, some authors contraindicate the use of implants in patients with osteoporosis, while others believe it is not a determining factor to contraindicate therapy with dental implants since the professional performs an appropriate treatment plan with modified geometry, larger diameter, implant surface treatment and a longer waiting period for prosthetic load application^13,14^.

Regarding to the impact of osteoporosis on dental implant survival, Chen, et al.^8^ (2013) and Busenlechner, et al.^5^ (2014) reported that since there is no statistically significant differences between osteoporotic and non-osteoporotic patients that had been rehabilitated with dental implants, there is a strong correlation between this bone metabolism condition and implant survival, mainly in the mandible. These studies did not include a feasible number of patients to establish a comparison between osteoporotic and non-osteoporotic patients. Besides that, these clinical studies did not show indicate drugs used for osteoporosis.

Currently, several therapies are available for the treatment of osteoporosis, however, they present some problems related to efficacy and long-term safety. The role of estrogen in bone integrity maintenance has been recognized for a while, although estrogen therapy shows several non-skeletal, adverse effects, including vascular events and breast carcinoma^5^_._ Some drugs act by decreasing bone resorption and therefore delaying bone loss rate (antiresorptive therapy), such as bisphosphonates, calcitonin and the human monoclonal antibody, denosumab, or by promoting bone formation (anabolic therapy), such as teriparatide^1,3,14,18,19,28^.

Sodium alendronate is part of the second generation of bisphosphonates, exhibiting less collateral effects in relation to the first generation and being the most used antiresorptive drug due to its low cost in comparison with other drugs^17^. The class of these drugs has a high affinity for human bone matrix and structural characteristics similar to pyrophosphate, that is, they are inhibitors of calcium hydroxyapatite crystal growth and exert their antiresorptive activity by inhibiting osteoclast development and migratory activity, and also by promoting their apoptosis^4,31^.

Several studies investigated the effects of the oral bisphosphonates in osteoporosis conditions related to the risk of fractures^1,4,6,14^, bone mineral density^3,6,9,20,28^, bone strength^3,28^ and bone repair^2,7,11,19^. These studies still remain without some information regarding the molecular and microstructural features around implants in osteoporotic situations. For this reason, this study through immunolabeling, polimerase chain reactions, and microtomography analysis aimed to evaluate the bone repair process at the bone/implant interface of osteoporotic rats treated with sodium alendronate.

## Material and methods

### Experimental groups

This study followed the standards of the Ethics Committee on Animal Use (2012/0109-6) of the Brazilian College of Animal Experimentation - COBEA. Forty-two rats (*Rattus norvegicus albinus,* Wistar) of approximately 200 grams were divided in to three groups: CTL (rats submitted to fictitious surgery and fed with a balanced diet containing 1.4% Ca, 0.8% P and water *ad libitum*); OST (rats submitted to bilateral ovariectomies and fed with a low calcium diet containing 0.1% Ca, 0.5% P and water *ad libitum*, without medical treatment); and ALE (rats submitted to bilateral ovariectomies, fed with a low calcium diet and treated with sodium alendronate).

For the quantitative analysis (micro CT and PCR), after the Power Test calculation, the sample number for each group was a minimum of 6 (power test=0.8). Thus, as PCR was made in quadruplicate, we elected 4 animals (left tibia) for this evaluation. For the micro CT evaluation, 6 animals were selected (4 right tibia belonging to the PCR groups plus 2 animals) with a total number of 18 animals.

For the qualitative analysis (immunhoistochemical), 4 animals *per* group were selected for the periods (14 and 42 days). Thus, we had obtained 24 animals. (N=42).

### Estrous cycle evaluation

The evaluation of the estrous cycle was performed according to the method described by Long and Evans^16^(1922), in which rats were separated into individual cages and their estrous cycles were assessed daily, for eight days.

### Bilateral ovariectomy and low calcium diet (osteoporosis induction)

All rats were initially anesthetized with xylazine hydrochloride (Dopaser - Laboratório Calier do Brasil, Ltd. - Osasco, SP, Brazil) and ketamine (Vetaset - Fort Dodge Animal Health Ltd, Campinas, SP, Brazil) to perform incisions on both flanks and to remove both ovaries from the rats of the OST and ALE groups. From the CTL group, only the ovary exposure was performed.

The rats from the OST and ALE groups were fed with a low calcium diet (containing 0.1% Ca, 0.5% P and water *ad libitum*) and the CTL group was fed with a balanced diet containing 1.4% Ca, 0.8% P and water *ad libitum*.

The low calcium and phosphate diet was used in order to simulate a real osteoporosis situation. According to previous studies^21,26,27^, when rats were subjected to bilateral ovariectomies and fed with low calcium diet, there was a decrease of bone mineral density up to two higher greater. Thus, only those undergoing ovariectomy surgery could have an osteopenia condition.

### Sodium alendronate

Eight days after ovariectomy, the drug therapy was initiated by gavage with 0.1 mg /kg/day of sodium alendronate dissolved in an aqueous solution to the ALE group, as designed by Paz, et al.^20^(2001), and gavage with saline solution to the OST and CTL groups. This treatment was conducted until the end of the experiment.

### Implants

After fasting for eight hours, the animals were sedated using 50 mg/kg of intramuscular ketamine (Vetaset - Fort Dodge Animal Health Ltd, Campinas, SP, Brazil) and 5 mg/kg of xylazine hydrochloride (Dopaser - Laboratório Calier do Brasil, Ltd. - Osasco, SP, Brazil). Furthermore, mepivacaine hydrochloride (0.3 ml/kg, scandicaine 2% with adrenaline 1:100,000, Septodont, Saint-Maur-des-Fossés, Paris, France) was used for analgesia and local vasoconstriction.

Then, the trichotomy was performed in the medial region of both tibias along with the antisepsis using Polyvinyl Pyrrolidone Iodine degermant (10% PVPI, Riodeine Degermant, Rioquímica, São José do Rio Preto, SP, Brazil) and a topical solution. An incision of approximately 3 cm was made with a divulsion of the soft tissue up to the tibial metaphyses exposure point.

In both tibias, a commercially available titanium implant with its surface treated by double acid etching was installed (Mater Pourus, Conexão Implant Systems Ltd., São Paulo, SP, Brazil). All implants were 1.6 mm in diameter and 3.0 mm in height. For milling, a spiral milling cutter with a diameter of 1.4 mm was used, mounted on an electric motor (BLM 600^®^; Driller, São Paulo, SP, Brazil) at a speed of 1000 rpm under irrigation with a 0.9% saline solution (Fisiológico^®^, Laboratories Biosintética Ltda^®^, Ribeirão Preto, SP, Brazil). The installation was manually conducted with a digital key.

After placement of the implant, the suture was performed with absorbable wire (Polyglactin 910 - Vycril 4.0, Ethicon, Johnson Prod, São José dos Campos, SP, Brazil) in the deep plan and with monofilament wire (Nylon 5.0, Ethicon, Johnson, Sao José dos Campos, SP, Brazil) on the external plan. Pentabiotic was administered (0.1 ml/kg, Fort Dodge Animal Health Ltd, Campinas, SP, Brazil) in a single intramuscular dose as was Sodium Dipyrone (1 mg/kg/day, Ariston Chemical and Pharmaceutical Industries Ltd., São Paulo, SP, Brazil) in the immediate postoperative period.

In order to collect material, the animals were anesthetized following the anesthesia protocol for implant placement. Then, the implants of left tibias were removed by counter-torque and the bone material that was previously in contact with the implant was collected for RT-PCR analysis (real time polymerase chain reaction). At this moment, the rats were euthanized as outlined by Ramalho-Ferreira, et al.^22^(2015) and the right tibias were removed and reduced with margins of about 1 cm to perform the Micro-CT analysis.

### Microtomography evaluation (Micro-CT)

For the three-dimensional analysis of animals from the CTL, OST and ALE groups after euthanasia, at the 42-day period, the right tibias that had been removed were fixed in formalin for 48 hours, washed for 24 hours and kept in 70% ethanol. These pieces were first examined by beam scanning X-ray in a digital computerized microtomography.

The pieces were scanned by a SkyScan microtomographer (SkyScan 1176 BrukerMicroCT, Aatselaar, Belgium, 2003), using sections that were 9 µm in thickness (80 Kv and 300 μA), with a copper-aluminum filter and a 0.3 mm rotation step, pixel size of 12.45 μm and an acquisition time of 1 h and 26 min. The images obtained by the projection of X-rays on the samples were stored and reconstituted to determine the area of interest by the NRecon software (SkyScan 2011, Version 1.6.6.0), with an artifact ring correction of 8, beam hardening correction of 24% and the image conversion varied from 0.0 to 0.14. Using the Data Viewer software (SkyScan, Version 1.4.4, 64-bit), the images were reconstructed and observed in three planes (transversal, longitudinal and sagittal). Then, the CTAnalyser-CTAn software (2003-11SkyScan, 2012 BrukerMicroCT Version 1.12.4.0) was used to determine bone volume, total volume of pore space and total porosity (percent), finally the 3D reconstruction was performed using the CTvox software (SkyScan, Version 2.7).

### RT-PCR

After removing the left tibial implants, the bone around the peri-implant defect was removed with a Carborundum disc mounted on a straight handpiece and micromotor. Pieces for molecular analysis were processed to perform the experiments in RT-PCR StepOne Plus, in order to evaluate the expression of the gene encoding proteins related to bone repair.

To execute the experiment, each bone fragment removed after material collection was carefully washed in a phosphate buffered saline solution (PBS), frozen in liquid nitrogen and stored at -80°C in a freezer for total RNA extraction with Trizol reagent (Life Technologies Invitrogen, Carslbad, CA, USA) and the SV Total RNA Isolation System extraction kit (Promega, Madison, Wisconsin, USA) according to the manufacturer’s specifications. After extracting total RNA from each sample, the quantification and analysis of concentrations and purity were performed using a spectrophotometer, and the integrity was analyzed by use of a denaturing agarose gel.

Following these analyzes, the normalization of total RNA concentration of each sample was performed with ultrapure water (Sigma-Aldrich, St. Louis, Missouri, USA) for the manufacturing of cDNA strands by the reverse transcriptase reaction, using the High Capacity cDNA Reverse Transcription kit (Applied Biosystems, Foster City, California, USA). The RT-PCR StepOne Plus reactions for the quantification of gene expression related to bone repair were made using SybrGreen system (Applied Biosystems, Foster City, California, USA) in the StepOne Plus device (Applied Biosystems, Foster City, California, USA). The reactions were performed in quadruplicate, Taqman Universal PCR Master Mix was added to plates containing the genes of interest and cDNA volumes were calculated in accordance with the samples quantification.

The amplification reaction was performed at 50°C for 2 minutes, 95°C for 10 minutes, with 40 cycles at 95°C for 15 seconds and then at 60°C for 1 minute (denaturation and extension). The results were analyzed based on the Ct value (cycle threshold), that is the point corresponding to the number of cycles where the sample amplification reached a threshold (measured between the fluorescence level of negative controls and the phase of the samples exponential amplification), which allowed for the quantitative analysis of the interested genes expression and the ones related on the plate. As an endogenous control, the expression of beta-actin constitutive gene was evaluated, which is used for the normalization of expression levels of the genes evaluated (RUNX2, alkaline phosphatase, BSP and osteocalcin).

### Immunohistochemistry

The pieces were fixed in formalin, washed under running water and decalcified in EDTA (10%). Then, dehydration was carried out using a sequence of alcohols. The diaphanization was performed with xylol for later inclusion in paraffin to obtain sections with thicknesses of 5 μm, and then mounted on slides. The immunohistochemical reactions were used to characterize the osteoblastic phenotype from the presence of proteins that featured different stages of osteoblast maturation, starting with the transcription factor RUNX2 (pre-osteoblast cells); Alkaline Phosphatase, showing the beginning of mineralization process by phosphate ion precipitation; Osteopontin, which marks mature osteoblasts and the beginning of bone mineralization activity; and Osteocalcin, which is a late protein, considered to be the marker of bone mineralization, representing the last stage of osteoblast maturation. These proteins were analyzed in periods of 14 and 42 days.

This process was obtained by inhibiting the activity of endogenous peroxidase with hydrogen peroxide, using primary antibodies against RUNX2, alkaline phosphatase, osteopontin and osteocalcin. These antibodies are polyclonal and are produced in goats (Santa Cruz Biotechnology, Paulinia, SP, Brazil).

Immunohistochemical experiments were performed using immunoperoxidase as detection medium. The antibody used was the secondary biotiniladoanti-goat produced in rabbits (Pierce Biotechnology, São Paulo, SP, Brazil), the amplifier was Streptavidin and Biotin (DAKO Japan, Tokyo, Japan), and Diaminobenzidine (DAKO Japan, Tokyo, Japan) was used as a chromogen. The proteins expression was analyzed in the region of the implant threads, by assigning scores: 1 representing light staining, 2 moderate and 3 severe. These scores were established according to the study performed by Ramalho-Ferreira, et al.^21^ (2016), in which light staining represented about 25% of the immunolabeling area in the blades; moderate staining represented about 50% of the immunolabeling area in the blades; and severe staining represented about 75% of the immunolabeling area in the blades.

### Statistical analysis

For statistical analysis, the software Sigmaplot 12.3 (Systat Software, Inc. SigmaPlot for Windows, San Jose, CA, USA) was used. The homoscedasticity analysis was performed by Shapiro-Wilk test in order to distinguish the parametric and nonparametric data. For analysis of the PCR parametric data, the total pore volume and the percentage of total porosity (micro-CT), the One-way ANOVA and Tukey post-tests were performed. For bone volume heterogeneous data (micro-CT), the Kruskal-Wallis test with Dunn’s post-test were applied. The significance level adopted was p<0.05.

## Results

### Micro-Ct

The pieces’ scans were made in the CTL, OST and ALE groups, 42 days after implant placement. Quantitatively, the median bone volume obtained in the CTL group was 0.0256 mm^3^, in the OST group it was 0.0276 mm^3^, with a considerable increase in the ALE group, showing 0.0497 mm^3^.

**Figure 1:**
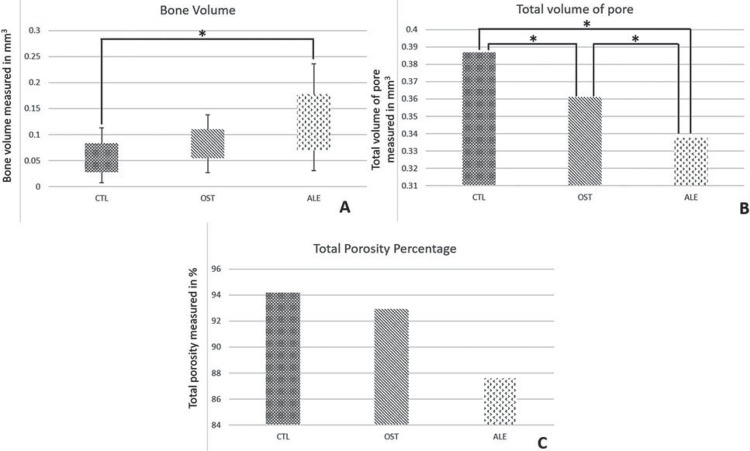
A- Graphic box-plot showing bone volume. CTL x OST x ALE: p>0.05. (*) p<0.05. B: Total volume of pore. p<0.05 (*). C: Total Porosity percentage. CTL x OST x ALE: p>0.05

Based on the results for bone volume, there were statistical differences between the 3 groups (p=0.012, Kruskal-Wallis), presenting a difference when comparing the CTL with the ALE group, with p<0.05 (Dunn’s post-test) (Figure 1A).

The averages of total pore volume in peri-implant bone tissue obtained for the CTL, OST and ALE groups were respectively 0.387, 0.361 and 0.338, in which only the ALE values showed statistical significance in relation to the others (p<0.05, Tukey test). Regarding the percentage of total porosity, the values were 94.189 (CTL), 92.924 (OST) and 87.596 (ALE) (p=0.084, Tukey test) (Figures 1B and C).

### PCR

**Figure 2:**
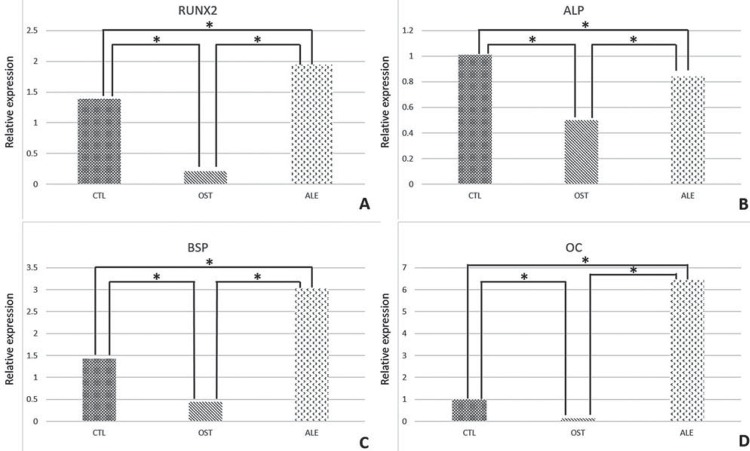
A- RUNX 2. (*) p<0.05. B: ALP. (*) p<0.05. C: BSP. (*) p<0.05. D: OC. p<0.05 (*)

The osteoblastic differentiation protein RUNX2 was significantly expressed in the ALE group (1.94), followed by the CTL (1.39) and the OST (0.21) (p<0.001, Holm Sidak) (Figure 2A).

Considering the ALP, the highest values were observed in the CTL (1.01), followed by the ALE (0.84), and the lowest values were exhibited by the OST group (0.50) (p<0.05, Tukey test) (Figure 2B).

The BSP protein was markedly expressed in the ALE group (3.03), followed by the CTL (1.43) and OST (0.44) groups (p <0.001, Holm-Sidak) (Figure 2C).

Concerning the osteocalcin, only the comparison between the ALE (6.43) and the OST (0.13) presented a statistical significance (p <0.05, Tukey test), with a tendency of higher values for the ALE group in relation to the other groups studied, including the CTL (1.00) (Figure 2D).

### Immunohistochemical analysis

**Figure 3:**
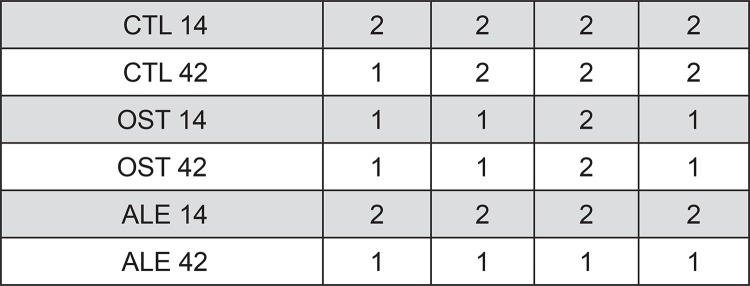
Scores observed in the marking of RUNX2, OP, OC and ALP proteins, in the different experimental groups

Immunohistochemical analysis performed at 14 and 42 days can be observed by the staining scores (Figure 3).

**Figure 4:**
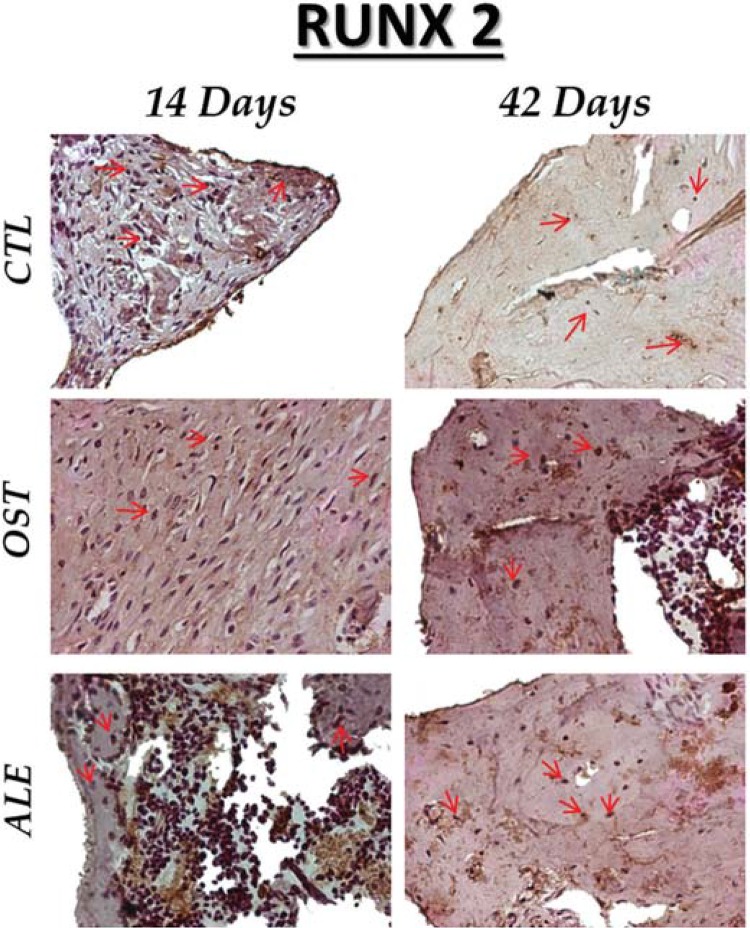
RUNX 2 immunostaining at 14 days for the experimental groups (CTL: moderate; OST: light; ALE: moderate). At 42 days (CTL: light; OST: light; ALE: light). Immunolabeling indicated by red arrows. (Original, 40x)

For RUNX 2, light to moderate staining was observed in the CTL group, light staining in the OST and moderate in the ALE at 14 days of osseointegration. After 42 days, immunostaining showed a light presence of RUNX2 in the three experimental groups (Figure 4).

**Figure 5:**
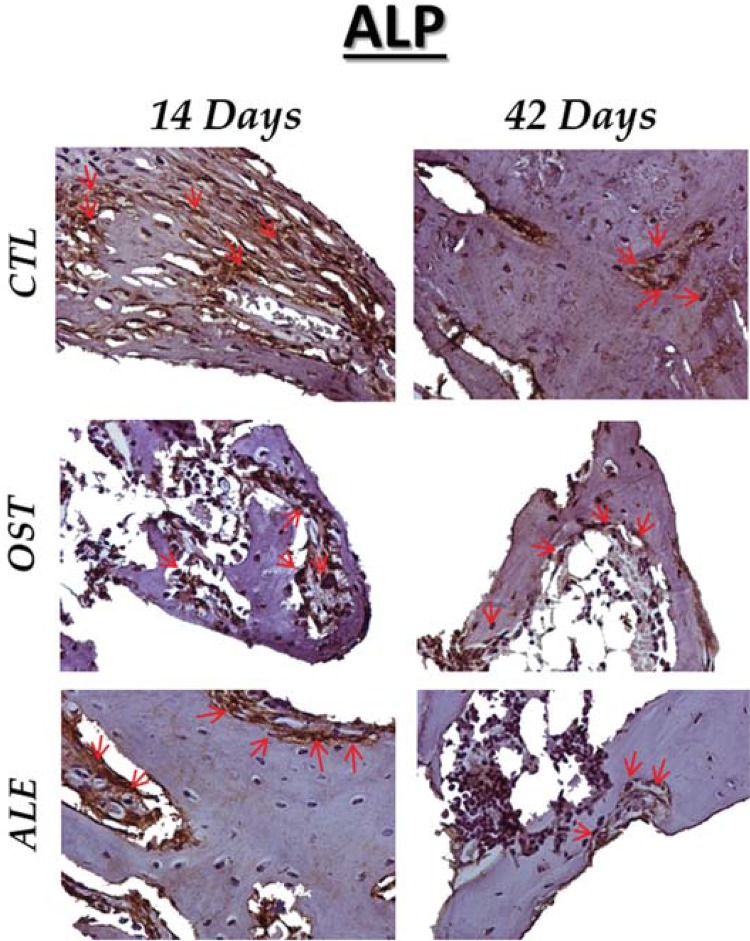
ALP immunostaining at 14 days for the experimental groups (CTL: moderate; OST: light; ALE: moderate). At 42 days (CTL: moderate; OST: light; ALE: light). Immunolabeling indicated by red arrows. (Original, 40x)

The alkaline phosphatase showed moderate expression in the CTL group at 14 and 42 days. Its reduction in the osteoporotic model (OST) is presented at 14 and 42 days, with light immunostaining. The administration of sodium alendronate in osteoporotic animals showed a moderate score at 14 days, with a reduction at 42 days (Figure 5).

**Figure 6:**
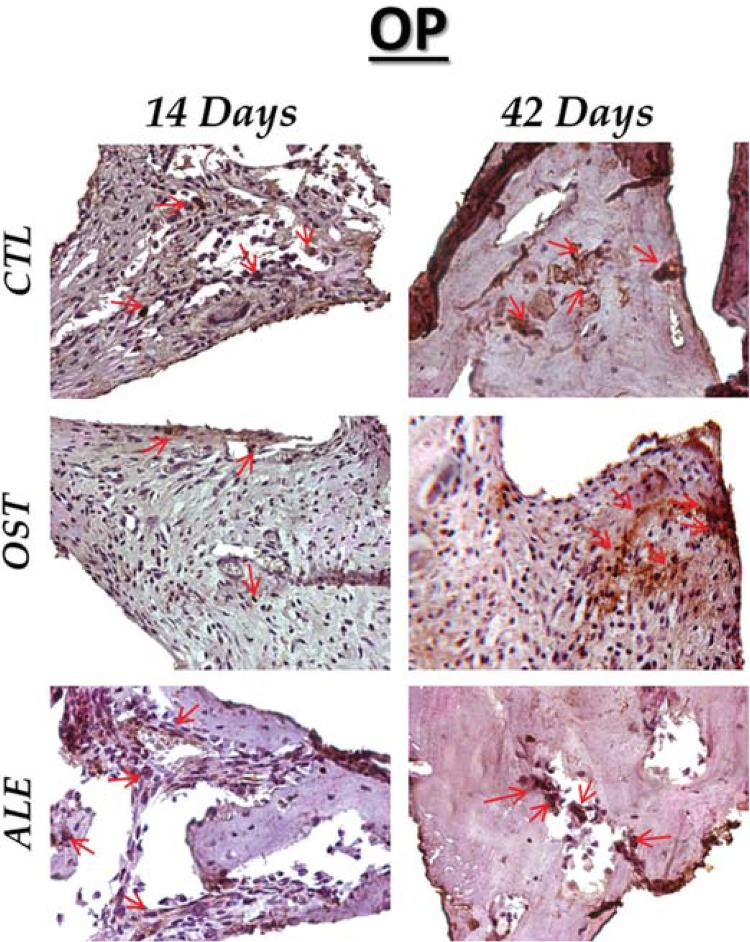
OP immunostaining at 14 days for the experimental groups (CTL: moderate; OST: moderate; ALE: moderate). At 42 days (CTL: moderate; OST: moderate; ALE: light). Immunolabeling indicated by red arrows. (Original, 40x)

The staining observed for osteopontin was moderate in the CTL, OST and ALE groups at 14 days of osseointegration. This moderate immunostaining was maintained at 42 days for both the CTL and OST groups, but became light in the ALE group (Figure 6).

**Figure 7:**
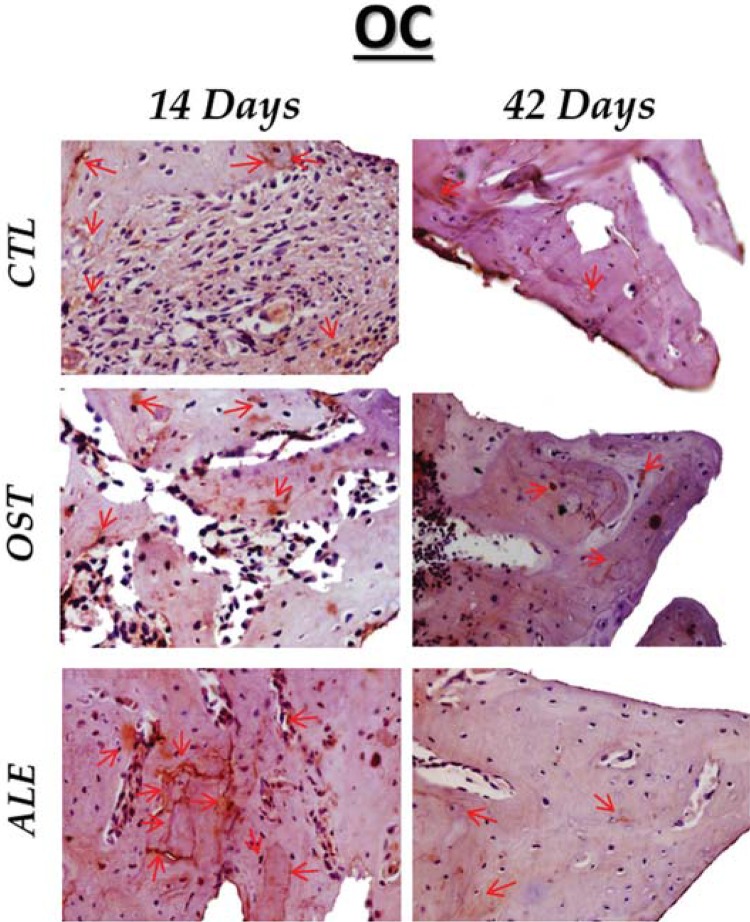
OC immunostaining at 14 days for the experimental groups (CTL: moderate; OST: light; ALE: moderate). At 42 days (CTL: moderate; OST: light; ALE: light). Immunolabeling indicated by red arrows. (Original, 40x)

Regarding osteocalcin labeling, moderate immunostaining was observed in the CTL group at 14 and 42 days, and in the OST and ALE groups, staining was moderate at 14 days, with mineral decrease indicated by a light immunostaining at 42 days (Figure 7).

## Discussion

The results of the current study were consistent to accept the hypothesis designed by the authors that the treatment with sodium alendronate in rats with induced osteoporosis would improve bone repair around the implants installed in the tibia. Through microtomographic (BV and Po.V) and molecular parameters (relative expression of RUNX2 and BSP) and through the proteins RUNX2 and OPN, it became evident that the rats from the ALE group exhibited superiority in repair standards for achieving greater bone volume and simultaneously a greater maturation in comparison to the control groups (CTL and OST). Except in phosphate precipitation in which the CTL was higher than the other groups.

A crucial factor for implant stability, especially in the early stages of the bone repair process, is the quality of tissue that will support the osseointegrated implant^29^. In this context, osteoporosis decreases bone mass in long bones and the maxillofacial complex, as demonstrated by clinical studies^15^. Considering that maxillary bones present decreased density due to the structural characteristics of their micro-architecture, mainly in the posterior regions, the presence of osteoporosis decreases the implants stability during installation and, therefore, increases the failure rate^9,25^.

Therefore, osteoporosis therapy consists of increasing bone mineral density in order to reduce long bone and vertebrae fractures. Thus, anti-resorptive medications, such as sodium alendronate, have been effective in this treatment^1,4,7,14,20^. Apparently, according to previous studies^7,11,12,19,28^, sodium alendronate is also able to improve bone repair at the bone/implant interface under osteoporosis conditions, at least at the beginning of the bone tissue evaluation.

Thus, the results of this study confirmed the anti-resorptive potential of ALE in lower quality bones. Considering the molecular and immunohistochemical results, the staining observed for RUNX2 showed lightly to moderately expressed pre-osteoblasts at 14 days in the OST group, presenting an increase in transcription factor expression, perhaps in an attempt to recruit a greater number of osteoblasts, offsetting the decrease in osteoblast activity due to the osteoporosis installed on this experimental group. The ALE group showed similar results to the CTL group, that is, moderate staining at 14 days, which confirms the beginning of mineralization, and light staining at 42 days, confirming the normal chronology of bone repair and showing a positive action of the administered medication. When quantifying this protein by real time PCR at 42 days, the highest values were found for the ALE group, even greater than the positive control group (CTL) (P<0.001), which demonstrated the highest signaling of osteoblast recruitment for bone tissue formation in this experimental group.

The immunostaining for osteocalcin, the marker of bone mineralization, showed differences in the quality of bone tissue formed during the repair process. The CTL group showed the highest expression of this protein, while the osteoporosis group presented the lowest expression. At 14 days, the presence of osteocalcin was similar in the ALE (moderate staining) when compared to the CTL group (moderate staining). However, at 42 days, it remained moderate in the CTL group and became light in the OST and ALE groups.

At 14 days, the bone lining cells were observed to have positive staining for this protein, especially in the CTL group. The moderate presence of osteocalcin in the CTL group at 42 days characterizes mature bones and a mineralization activity, which may be related to a satisfactory osseointegration process, featuring bone tissues that can offer adequate support for implant installation. However, at 42 days, the osteoporotic animals treated with alendronate showed slight staining for osteocalcin, characterizing a lower mineralization of the bone formed along the implants. These findings were consistent with molecular results, since even with a trend of increased gene expression for osteocalcin in relation to the other groups, there was no statistically significant difference (P>0.05). Hence, in the long term, ALE will probably present reduction in bone tissue maturation.

Whilst considering bone tissue maturation process, the reduction of alkaline phosphatase due to osteoporosis seems to be consistent with a decrease in bone quality in this systemic interference. Alkaline phosphatase is an enzyme which acts at the beginning of the phosphate precipitation process over the collagen matrix of bone tissue^31^. It is also considered to be a marker protein of the osteoblastic phenotype^10^. Studies have shown that the use of sodium alendronate, in the long term, can lead to alterations in this protein’s activity^24^. This drug has structural characteristics similar to pyrophosphate, inhibiting the growth of calcium hydroxyapatite crystals, with the increase in pyrophosphate being related to decreased mineralization. Because of this correlation, the immunostaining of alkaline phosphatase was important during the osseointegration periods in this study.

Some proteins like alkaline phosphatase are historically marked through serum concentration to detect and follow the course of hepatobiliary and skeletal disease. Whyte^31^ (1994) related that molecular studies of hypophosphatasia, a rare heritable form of rickets, have confirmed the long-held notion that ALP has a significant role in skeletal mineralization in humans. Thus, proteins linked to bone repair such as those used in this study (ALP, BSP, osteopontin and ostecalcin) through molecular and immunohistochemical reactions are very important in understanding the aspects involved with bone formation at different phases (osteoblasts induction and bone maturation).

Osteoblasts presented positive staining for osteopontin especially in regions close to implant spirals, showing activity of bone remodeling close to the osseointegration areas. Staining for osteopontin was moderate in the CTL, OST and ALE groups at 14 days of osseointegration due to the intense cellular activity in this period. This moderate immunostaining remained at 42 days for both the CTL and OST groups, but became light in the ALE group. It is worth noting the presence of bone lining cells, positive for osteopontin in the CTL group at 14 days, showing the formation of the osteopontin interfaces.

Tomographic parameters are essential for the correct planning of the clinical atrophy diagnosis of a bone that will receive implants, because of the cortical/medullary characteristics observed in more accurate imaging exams, which will influence the choice of the implants macrogeometry, the best surface treatment technique and the most indicated prosthetic connection^2,11^. The microtomography performed on the pieces of this study was directly related to the increase in bone volume to ALE, even in comparison with CTL (p=0.012), at the same time in which the characteristics related to density, the ALE group presented smaller pore volumes than the other groups (CTL and OST; p>0.05). These characteristics indicate that treatment with ALE promoted higher volumes of available bone and higher densities, fundamental factors for the stability of rehabilitation treatment.

Even though these results are encouraging for the primary indication of alendronate under these osteoporotic conditions, especially regarding the best structural bone characteristics, an evident concern is about maintaining these characteristics over time. The molecular and immunohistochemical data showed a reduction in bone mineralization markers and, consequently, a cellular characteristic that is unfavorable to the process of bone tissue formation in treatments with alendronate. These results suggest that in the long term, there is a detriment in the quality of bone formed during the osseointegration process, which seems to be correlated with the reduced production of the proteins that favor bone tissue formation.

In addition to these probable limitations, the literature also describes the possibility of this treatment to cause maxillary osteonecrosis^23^. Even though alendronate is an oral bisphosphonate that is less powerful when compared to other intravenous applications, the ALE is still closely linked to osteonecrosis induction, both in tooth extraction surgery and in loss of dental implants that have been, sometimes, installed for decades^17,31^.

This study was statically conducted on peri-implant bone, although it is known that the greatest problem is related to dynamic changes in the bone, mainly due to physiological changes observed during mastication loads on implant-supported prostheses. Given the statements above, new studies must be designed, with a primary transcutaneous load application on the implants after bone tissue maturation (42 days), with micromotion devices, as described by Wazen^30^ (2013).

As previously emphasized, another issue to be investigated concerns the question: what is the behavior of osteoporotic bone treated with ALE in the long term? Thus, future research should examine the same conditions of this study, but at 6 month and 1 year periods after implant placement, to mimic rehabilitation after 24 and 48 months, corresponding to humans.

Based on the information described above and on the limits of this *in vivo* study, it was concluded that short-term treatment with sodium alendronate in rats with induced osteoporosis improved bone repair around the implants installed in the tibia. Otherwise, more studies are necessary in order to evaluate the quality of the peri-implant bone under long-term sodium alendronate administration in the presence of the osteoporotic condition.
